# Psychological impact of COVID-19: A cross-lagged network analysis from the English Longitudinal Study of Aging COVID-19 database

**DOI:** 10.3389/fpsyt.2023.1124257

**Published:** 2023-02-22

**Authors:** Cristian Ramos-Vera, Angel García O'Diana, Miguel Delgado Basauri, Dennis Huánuco Calle, Jacksaint Saintila

**Affiliations:** ^1^Research Area, Faculty of Health Sciences, Universidad César Vallejo, Lima, Peru; ^2^Sociedad Peruana de Psicometría, Lima, Peru; ^3^Postgraduate School, Universidad Femenina del Sagrado Corazón, Lima, Peru; ^4^Escuela de Medicina, Universidad Señor de Sipán, Chiclayo, Peru

**Keywords:** anxiety, comorbidity, COVID-19, depression, mental health, loneliness

## Abstract

**Background:**

The COVID-19 pandemic and its subsequent health restrictions had an unprecedented impact on mental health, contributing to the emergence and reinforcement of various psychopathological symptoms. This complex interaction needs to be examined especially in a vulnerable population such as older adults.

**Objective:**

In the present study we analyzed network structures of depressive symptoms, anxiety, and loneliness from the English Longitudinal Study of Aging COVID-19 Substudy over two waves (Months of June–July and November–December 2020).

**Methods:**

For this purpose, we use measures of centrality (expected and bridge-expected influence) in addition to the Clique Percolation method to identify overlapping symptoms between communities. We also use directed networks to identify direct effects between variables at the longitudinal level.

**Results:**

UK adults aged >50 participated, Wave 1: 5,797 (54% female) and Wave 2: 6,512 (56% female). Cross-sectional findings indicated that difficulty relaxing, anxious mood, and excessive worry symptoms were the strongest and similar measures of centrality (Expected Influence) in both waves, while depressive mood was the one that allowed interconnection between all networks (bridge expected influence). On the other hand, sadness and difficulty sleeping were symptoms that reflected the highest comorbidity among all variables during the first and second waves, respectively. Finally, at the longitudinal level, we found a clear predictive effect in the direction of the nervousness symptom, which was reinforced by depressive symptoms (difficulties in enjoying life) and loneliness (feeling of being excluded or cut off from others).

**Conclusion:**

Our findings suggest that depressive, anxious, and loneliness symptoms were dynamically reinforced as a function of pandemic context in older adults in the UK.

## Introduction

In 2020, the UK was one of the countries most affected by the pandemic. Despite the existence of cases since the last days of January, the British government released the state of containment by the end of March as they sought other alternatives such as acquiring herd immunity in the population (1). Along with these measures, the British government sought to be among the first to implement the COVID-19 vaccines, being distributed to high-risk groups such as older adults, their caregivers, and health care workers in early December (2). However, from September to December there was an increase in the number of infections due to a new strain of COVID-19, prompting the UK government to introduce social restriction measures to control the second wave outbreak (3). These measures involve continuing the state of quarantine, forced isolation, and lockdown in most European countries and around the world, as was the case during the first wave that lasted until the end of July (2).

Loneliness is defined as an unpleasant subjective perception resulting from poor interpersonal relationships, both in terms of the number of friends or companions, as well as the quality of the social ties established, so that the latter makes it easier to differentiate from objective isolation (4). The psychological effects of confinement during COVID-19 pandemic are still being studied, but some findings so far are concerning. Several systematic and longitudinal studies have linked the state of confinement to negative psychological effects such as stress, anxiety, and depression (5–8). Older adults are particularly vulnerable to these effects given their higher risk of contagion and fear of infection and death (9, 10). Additionally, home quarantines can lead to feelings of fear, insomnia, post-traumatic stress disorder, or distress in the absence of social connection and perceived social support, combined with the pandemic context of increased uncertainty and fatalistic news (11).

Depression is defined as an affective disorder that reduces vitality and involvement in activities that previously produced pleasure, accompanied by extreme tiredness, discouragement, and loss of meaning in life (12). Anxiety is an adaptive response that involves physiological and cognitive symptomatology to cope with states of restlessness and agitation in response to real or imagined danger (13). Both anxiety and depression are considered as responses to stressful events that produce biological and psychological changes in the organism (14). These disorders are considered the most common during old age because as time goes by and throughout the aging process people lose mobility, chronic and/or degenerative diseases appear, they show greater fragility and require the care of other people, which triggers negative emotional states (15, 16). This, coupled with higher levels of loneliness due to home quarantine in older adults, reinforces a higher degree of psychological vulnerability (17, 18).

A survey conducted in the UK during the onset of confinement revealed that 22.1% of subjects had depression and 21.6% had generalized anxiety, where these were the most common symptoms affecting mental health due to the COVID-19 outbreak (6). There is a strong correlation between the two variables because they share similar indicators such as loss of control, difficulty concentrating, and fear (19, 20). Generalized anxiety disorder implies a continuum with depression, such that people with this comorbidity will experience greater functional disability, worse prognosis and persistence of other diseases (8).

Recently, some authors have begun to reexamine psychopathology from a more dynamic perspective to explain that psychological constructs may be conceived as a network of multiple symptoms correlated with each other (21). A psychometric network consists of a set of nodes represented as psychological symptoms and edges for the relationship between such variables. Network analysis is a tool that facilitates the understanding of different psychological variables, their comorbidities, possible risk and protective factors, as well as the most central symptoms within the network structure (22). Thus, it is necessary to use complex interaction systems to examine the most significant symptoms and signs that are affected by the outbreak of COVID-19 and social isolation.

Although unpleasant feelings of isolation and lack of meaningful connections are known to be related to depressive and anxious symptoms, it is not known exactly in which direction these interconnected associations may occur in a network system, given the period of the first and second COVID-19 quarantine (11). Based on these periods of change during the pandemic, it is appropriate to examine this associative system of psychopathological variables using longitudinal network analysis. This perspective extends the application of other traditional network analysis methods by detecting complex and dynamic interactions across two or more temporal events through directed networks between symptoms (23); i.e., it allows to represent and identify those symptoms that are more predictive in a network based on established time points, so that it is possible to distinguish the symptoms that reinforce each other and maintain a certain symptomatology (24).

Given these characteristics, it may be applicable to understanding the variation in emotional and/or psychophysiological responses during different periods of COVID-19 quarantine in the UK. Therefore, the aim of this study was to examine and compare symptom networks of loneliness, depression, and anxiety in two specific quarantine periods in a population of older adults in the United Kingdom. Likewise, in accordance with previous findings on the overlapping nature of the variables (25, 26), we use the Clique Percolation Method (CPM) to examine symptoms that may belong to more than one community or overlap with it. From this, we sought to identify the most central measures that would allow the understanding of indirect associations between systems. Finally, we examined a longitudinal network structure that allowed us to represent the prediction and feedback between symptoms that may trigger certain dysfunctional symptomatological patterns manifesting in older adults during two critical periods of mandatory 2020 quarantine.

## Materials and methods

### Participants

We analyzed data from the English Longitudinal Study of Aging (ELSA) COVID-19 Substudy, a national survey that assessed variables related to physical and mental health in people over 50 years of age in the United Kingdom during periods of strict COVID-19 quarantine. The study collected data on the health impact of COVID-19 in two stages: between June and July 2020 (Wave 1) and between November and December 2020 (Wave 2).

In the present investigation, we analyzed symptoms of depression, anxiety, and loneliness, which were assessed by online questionnaires and computer-assisted telephone interviews. All participants agreed to be part of the study after being informed of the study objective and informed consent. Initially, the data consisted of 7,040 and 6,794 responses in the first and second waves, respectively. After filtering for inaccurate or missing data, the total number of participants in the first period was 5,797, while in the second stage it was 6,512 older adults.

### Data analysis

#### Packages

All data and analyzes was processed with R language (27) in the R Studio IDE (27), using the following packages: qgraph (28, 29), igraph (30, 31), bootnet (32), glmnet (33–35), CliquePercolation (36), networktools (37), NetworkComparisonTest (38), and lavaan (39).

#### Contemporaneous networks

To identify redundant items, we conducted a Goldbricker analysis. The results indicated that there was no significant overlap between any of the items related to anxiety, depression, or loneliness. This allowed us to say that, although there are similar items in content, they measured different things, for example, “feeling isolated” which can be associated with feeling disconnected from others, while “feeling lonely” can refer to an emotional state of sadness due to a lack of company.

The partial correlation network structure for both models was calculated with bootnet package (32), through huge estimator (40, 41), a non-paranormal conversion of the data (42), and the rotation information criterion (ric) were used for model selection (43). The communities were explored using the spinglass clustering algorithm (44–46) through 500 spins at both times.

Then, an analysis of overlapped community was conducted with the cpAlgorithm function in the CliquePercolation package (36), percolated items were founded through the weighted CFinder method (47) by a *k* = 4 cliques and an intensity of *I* = 0.08, for two-waves networks.

The centrality was explored by the Expected Influence one-step (EI1; i.e., sums of the edge weights at directly related nodes), and two-step (EI2; i.e., sums of the edge weights at indirectly related nodes) (48), also the Bridge Expected Influence one-step (BEI1; i.e., sum of edge weights that connect each node with nodes of other communities directly related) and two-step (BEI2; i.e., sum of edge weights that connect each node with nodes of other communities indirectly related) (49).

Finally, two-time networks were compared with the “NCT” function of the NetworkComparisonTest package (38), with a bonferroni-holm correction technique (50, 51), and 1,000 permutations. The similarity of the networks was explored through correlation of the adjacency matrices and its centrality indices, if the result is 1 the networks have a perfect linear relationship, which means that networks have the same structure; if the correlation coefficient is 0, networks have no detectable linear correspondence; and if correlation coefficient is −1, networks are exact opposites (52).

#### Temporal network

The temporal network from a Cross-Lagged Panel Network (CLPN) approach where the relations among individual items are modeled, both within a time point and across time. This was applied into two steps. First: Fit a series of regularized regression models to estimate the cross-lagged and auto-regressive coefficients across time. Second: Summarize the results by producing plots and computing summary statistics such as nodewise in-prediction and nodewise out-prediction (53).

To estimate linear regression coefficients of each variable at T2 on itself and all other variables at T1, we used penalized maximum likelihood with a lasso penalty (33). This shrinks all small regression paths to exactly zero, while making the other paths larger. The result is a sparse network, in which many of the paths from variables at T1–T2 will be estimated as exactly zero. The regularized regression estimates were obtained for a sequence of 100λ values, and the one that produces the lowest cross-validation error is chosen for the final model.

Then, the in- and out- predictions of the network were estimated, like two centrality measures, the first (in-prediction) is the extent to which each variable is predicted by other network variables. This can be obtained through the proportion of variance in each variable at T2 that is explained by the full set of variables at T1, which can take values from 0 to 1. The second (out-prediction) is the extent to which each variable at T1 predicts other network variables at T2. This can be computed as a sum of squared outgoing standardized regression coefficients of the target variable at T1 predicting each variable at T2.

All the procedure and codes can be revised in Rhemtulla et al. (53).

#### Instruments

##### Center for Epidemiological Studies-Depression Scale

Depression was measured with the Center for Epidemiological Studies-Depression Scale (CES-D) developed by Radloff (54) in its 7-item version (e.g., “Much of the time during the past week: felt sad”) under a unidimensional model. It aims to recognize depressive symptomatology within the last week, with a Likert-type response scale of four alternatives ranging from 0 to 3 points (rarely, sometimes, a moderate amount of time, most of the time). The minimum score is 0 and the maximum score is 21.

##### Generalized Anxiety Disorder scale

To measure anxiety, the Generalized Anxiety Disorder scale (GAD-7) created by Spitzer et al. (55) was used to detect the severity of generalized anxiety symptomatology according to DSM-V criteria over the last 2 weeks. It consists of seven items (e.g., “Over the last 2 weeks: Feeling nervous, anxious or on edge”) within a unidimensional model, with a Likert-type response mode ranging from 0 to 3 points (never, several days, half of the days, and almost every day). The total scale has a minimum score of 0 and a maximum of 21.

##### University of California, Los Angeles Loneliness Scale

Loneliness was assessed with the University of California, Los Angeles Loneliness Scale (UCLA) created by Russell (56) with the objective of knowing the levels of loneliness. The three-item version was used (e.g. “How often feel: isolated from others”) constituted under a unidimensional model. The response rating scale ranges from 1 to 4 (never, rarely, sometimes, and often), where the total score maintains a minimum value of 3 and a maximum of 12 points.

## Results

Table 1 shows the items at the descriptive level, where the depressive item of feeling lonely (D5) stands out with the highest score (*M* = 1.843, SD = 0.364), followed by depressed mood (D1; *M* = 1.84.5, SD = 0.371). The highest and lowest skewness and kurtosis values correspond to the items of difficulty in resting (A5) and feeling lonely (D5), respectively. On the other hand, in the item reliability assessment, the item-rest correlation indices proved to be >0.30, as expected (57).

**Table 1:** Descriptive analysis of items.

**Item**	**Wave 1 (*****N*** = **5,797)**					**Wave 2 (*****N*** = **6,512)**				
	**Mean**	**SD**	**Skewness**	**Kurtosis**	**Item-rest correlation**	**Mean**	**SD**	**Skewness**	**Kurtosis**	**Item-rest correlation**
A1	1.493	0.773	1.713	2.574	0.777	1.574	0.829	1.501	1.653	0.784
A2	1.343	0.687	2.291	5.168	0.77	1.444	0.761	1.845	2.928	0.795
A3	1.472	0.743	1.715	2.696	0.781	1.559	0.798	1.522	1.901	0.797
A4	1.47	0.76	1.752	2.699	0.743	1.533	0.804	1.583	1.962	0.758
A5	1.335	0.689	2.311	5.135	0.611	1.351	0.713	2.237	4.61	0.637
A6	1.482	0.706	1.583	2.518	0.598	1.533	0.758	1.511	2.025	0.622
A7	1.343	0.669	2.24	5.089	0.693	1.383	0.712	2.072	4.051	0.716
D1	1.835	0.371	−1.803	1.251	0.613	1.813	0.39	−1.604	0.574	0.621
D2	1.781	0.414	−1.357	−0.157	0.591	1.75	0.433	−1.154	−0.668	0.623
D3	1.558	0.497	−0.233	−1.946	0.345	1.526	0.499	−0.103	−1.99	0.38
D4	1.814	0.389	−1.613	0.603	0.576	1.845	0.362	−1.908	1.641	0.573
D5	1.843	0.364	−1.888	1.566	0.471	1.823	0.381	−1.695	0.874	0.476
D6	1.18	0.384	1.669	0.784	0.598	1.82	0.384	−1.669	0.784	0.608
D7	1.787	0.409	−1.403	−0.03	0.564	1.702	0.457	−0.885	−1.218	0.576
L1	1.367	0.587	1.359	0.811	0.726	1.391	0.607	1.296	0.597	0.741
L2	1.332	0.543	1.38	0.944	0.71	1.34	0.55	1.363	0.893	0.707
L3	1.419	0.61	1.164	0.29	0.708	1.454	0.629	1.065	0.041	0.712

A1–A7, GAD-7 Scale items; D1–D7, CES-D Scale items; L1–L3, UCLA Loneliness Scale items.

**Figure 1:**
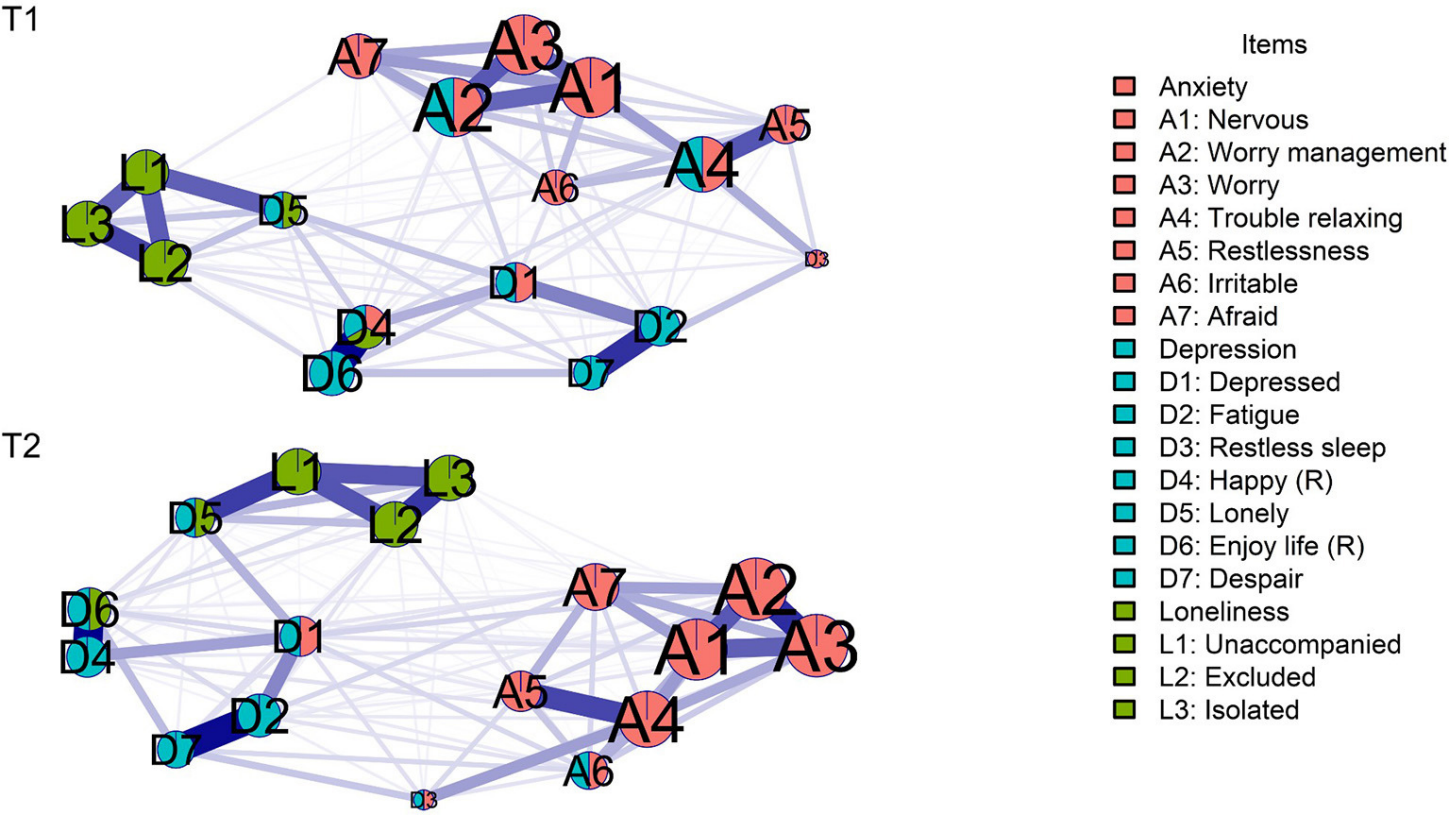
Contemporaneous network at both waves. Red cluster: anxiety; blue cluster: depression; green cluster: loneliness. Percolated nodes show 2 or 3 colors. Node sizes are predictability of each node. Reverse items are marked with (R).

In the two-waves contemporaneous network models (Figure 1), positive relationships between nodes were reported, where the relation between unhappiness (D4) and not enjoying life (D6; T1 *r* = 0.433; T2 *r* = 0.393) was the higher value, while the relation between trouble with relaxing (A4) and feeling excluded (L2; *r* = 0.0005) in time 1 (T1), and restless sleep (D3) with not enjoying life (D6; *r* = 0.002) in time 2 (T2) were the lowest values respectively. The explored clusters showed a coherent partitioning, while the percolated communities show that in Time 1 (T1), worry management (A2), trouble with relaxing (A4), and depressed mood (D1) items were overlapped by anxiety and depression communities; while the item of feeling lonely (D5) was overlapped by depression and loneliness communities; and unhappiness (D4) was overlapped by anxiety, depression and loneliness communities. Furthermore, in Time 2 (T2), feeling irritable (A6), depressed mood (D1), and restless sleep (D3) were overlapped by anxiety and depression communities, while feeling lonely (D5) and not enjoying life (D6) were overlapped by depression and loneliness communities. The CS-coefficient at time 1 (T1) and time 2 (T2) was CS = 0.75 for edge and strength stability.

At T1 the item of feeling nervous (A1) had the higher predictability (*r*2 = 0.60), while restless sleep (D3) had the lowest value (*r*2 = 0.18). Furthermore, at T2 the item of worry (A3) showed the higher predictability (*r*2 = 0.62), and restless sleep (D3) showed the lowest value (*r*2 = 0.20).

The network comparison test between T1 and T2 networks showed statistically significant differences only between the centrality indices of networks (*p* < 0.001); also, the adjacency matrices correlation coefficient showed linearity (rho = 0.86; *p* < 0.001), and in all of its centrality indices (*p* < 0.001).

**Figure 2:**
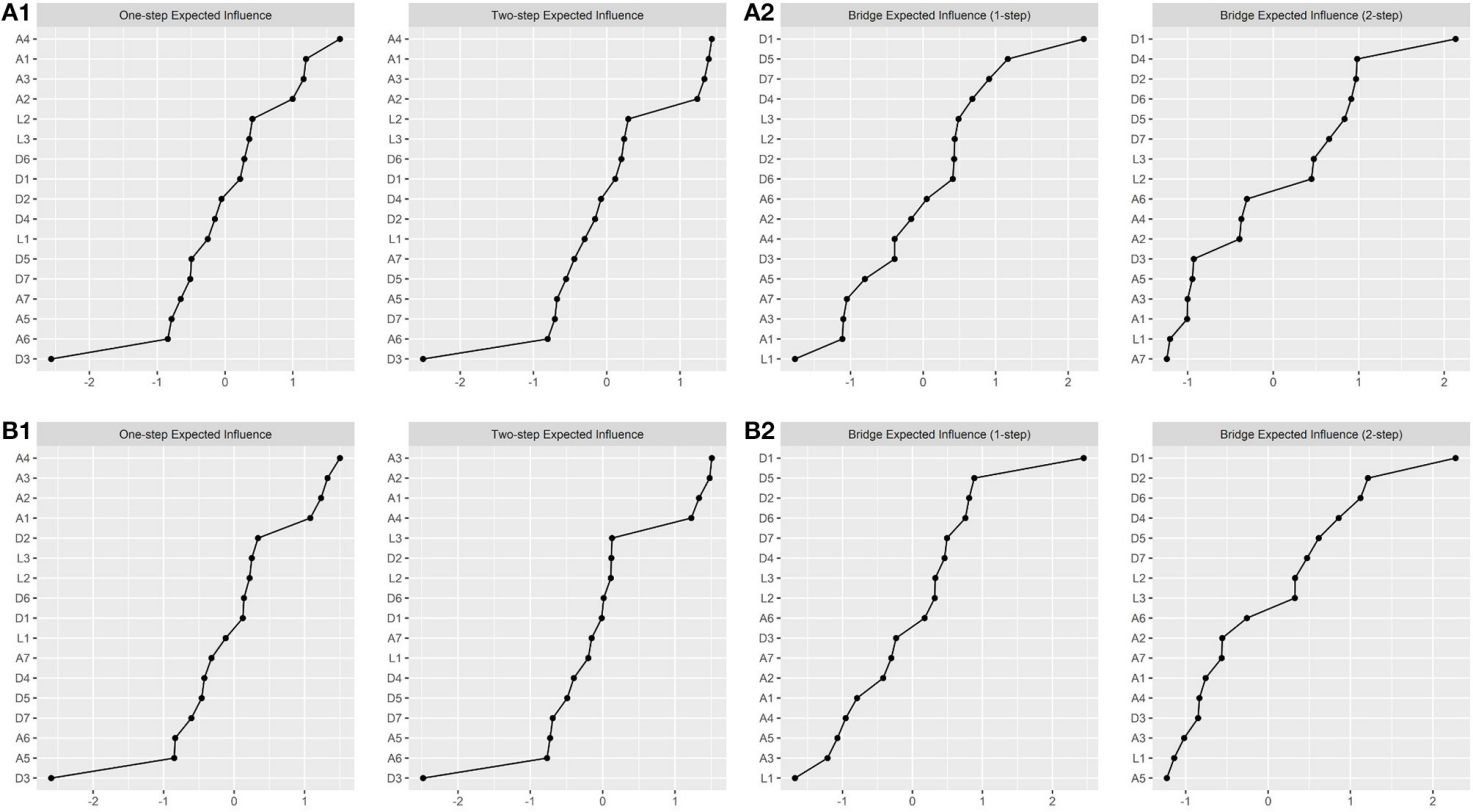
Centrality Indices for contemporaneous networks. **(A1)** First wave expected influence two steps; **(A2)** first wave bridge expected influence two steps; **(B1)** second wave expected influence two steps; **(B2)** second waves expected influence two steps. Values were scaled in *z*-scores.

Additionally, centrality indices are shown in Figure 2. At Time 1 the most central item was trouble with relaxing (A4; T1; EI1 = 1.162; EI2 = 2.152), and the lowest was restless sleep (D3; T1; EI1 = 0.479; EI2 = 0.932); while the higher bridge centrality was the item of depressed mood (D1; T1; BEI1 = 0.449; BEI2 = 0.901), the lowest bridge centrality 1-step was feeling unaccompanied (L1; BEI1 = 0.034) and bridge centrality 2-step was being afraid (A7; BEI2 = 0.293). On the other hand, at Time 2, some differences were founded, the highest one-step centrality was trouble with relaxing (A4; EI1 = 1.127) and two-step centrality was worry management (A2; EI2 = 2.170); the lowest centrality was restless sleep (D3; T2; EI1 = 0.499; EI2 = 0.974); while the highest bridge centrality 1-step was the depressed mood item(D1; T2; BEI1 = 0.478; BEI2 = 0.940) and the lowest were, in 1-step, feeling unaccompanied (L1; BEI1 = 0.057), and in 2-step, restlessness (A5; BEI2 = 0.315).

**Figure 3:**
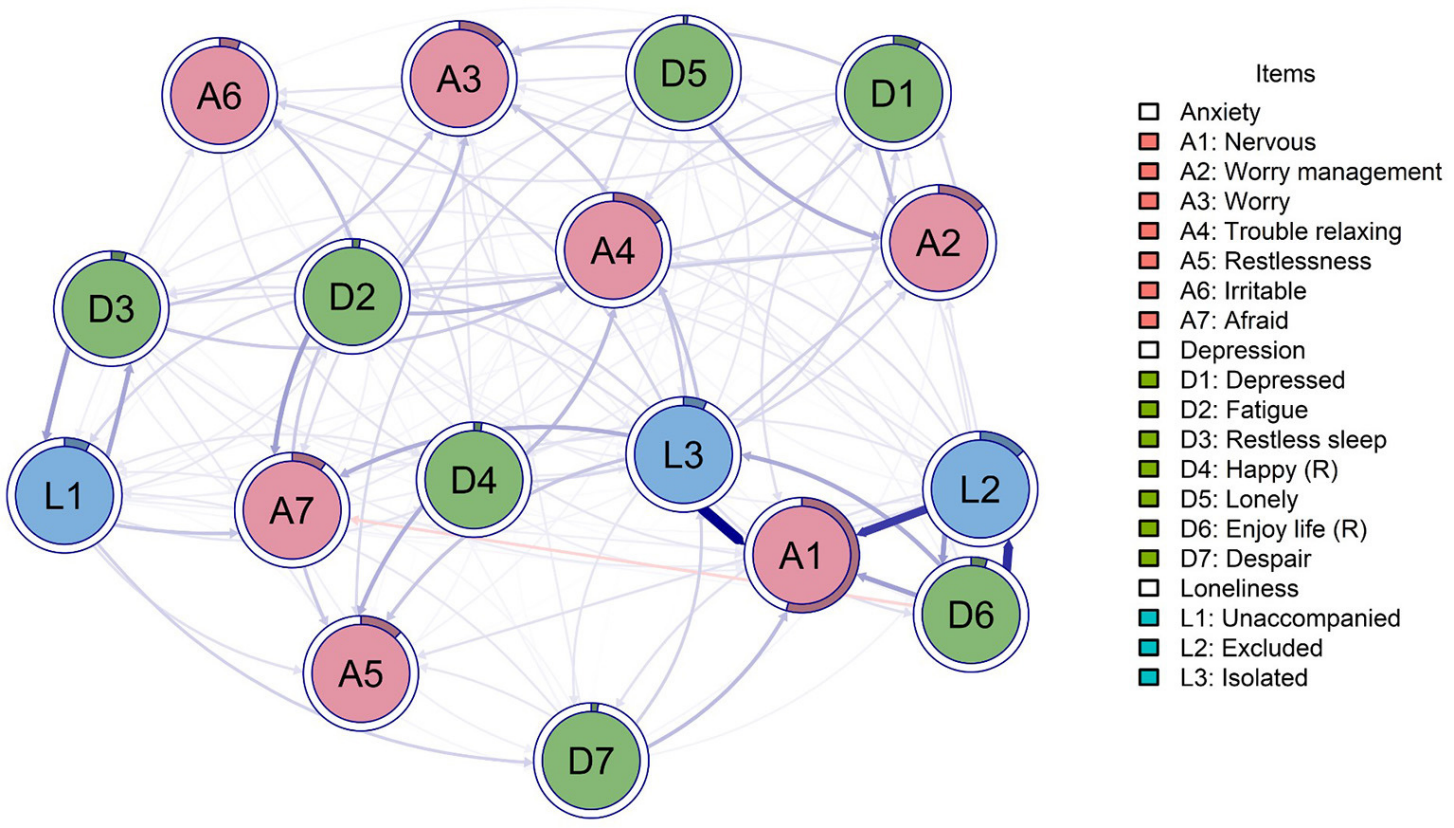
Cross-lagged network. Red cluster: anxiety; blue cluster: depression; green cluster: loneliness. Percolated nodes show 2 or 3 colors. Node ring is the in-prediction value of each node. Reverse items are marked with (R).

The temporal network shows cross-time effects from T1 to T2, most of the relationships were positive, except for being afraid (A7) predicted by not enjoying life (D6) that had a negative effect (*β* = −0.060). The highest relationships were on feeling isolated (L3) to feeling nervous (A1; *β* = 0.370), not enjoying life (D6) to feeling excluded (L2; *β* = 0.304), and feeling excluded (L2) to feeling nervous (A1; *β* = 0.284). In addition, all variables were autoregressive, except feeling nervous (A1), which did not show this characteristic, while feeling excluded (L2; *β* = 0.440) and not enjoying life (D6; *β* = 0.406) were the most autoregressive nodes. Furthermore, the regression model had excellent fit indices (CFI = 0.965; TLI = 0.965; SRMR = 0.010; RMSEA = 0.038) (Figure 3).

**Figure 4:**
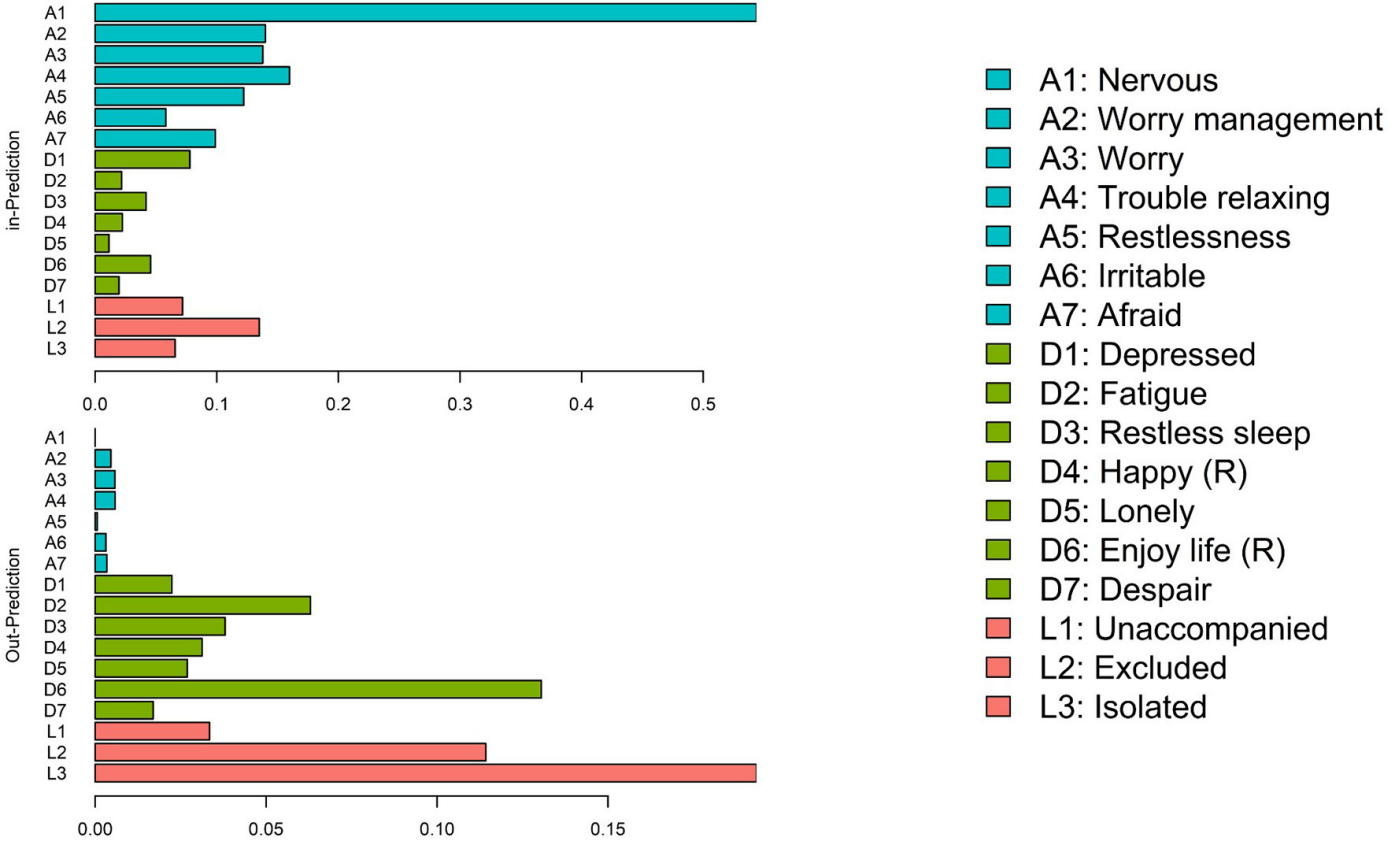
In- and out-prediction plot. Blue group: anxiety; green group: depression; red group: loneliness. Reverse items are marked with (R).

Finally, feeling nervous (A1) was the item more predicted at T2 (inPred = 0.544), and feeling isolated (L3) was the least predicted at T2 (inPred = 0.193). Furthermore, feeling isolated (L3) was the most predictive item at T1 (outPred = 0.193), while feeling nervous (A1) was the less predictive item at T1 (outPred = 0.000). This configures a direct predictive line from the feeling of loneliness to the enhancement of anxiety symptoms such as nervousness (Figure 4).

## Discussion

The United Kingdom was one of the countries with the highest number of infections during the beginning of the first wave of COVID-19 because the authorities expected herd immunity to develop in its entire population; however, as the number of infected people increased, they imposed a state of mandatory social isolation by the end of March as a control measure (1). During the months of September to December, a second wave occurred as a result of a new variant of the virus that led to a state of confinement (3). Of all age groups, older adults experience the greatest risk and vulnerability to COVID-19 symptoms due to the comorbidities of advanced age (58). They were also exposed to high levels of worry, anxiety, distress, sleep difficulties, feelings of loneliness, and depressive symptomatology due to fear of contracting the virus, fear of death, and involuntary social isolation (9, 10, 59, 60).

Regarding the first objective of the study, the estimated network during the first evaluation stage (June–July 2020) showed that anxiety symptoms were the most important (higher centrality), where item A4 (difficulty to relax) and item A1 (nervous or anxious) stood out. These results could be attributed to a stronger connection with the somatic symptoms of restlessness (A5) and generalized fear (A7). This could lead to an increased state of nervousness and difficulty relaxing in response to the rise in infections, contributing to higher levels of anxiety due to the increase in infections, hospitalizations and deaths due to COVID-19 (61), which is greater given the perception of belonging to an at-risk and more vulnerable population. Difficulty relaxing (A4) has been one of the most influential symptoms in previous network research involving symptoms of anxiety and depression during the initial pandemic stage of 2020 in adults in Asia, North America, and Europe (62–65). This finding was similar also in another study in UK adults during the first quarantine social restriction, which included overlapping symptoms of distress and loneliness (26).

As for the network analysis with the second wave data (November–December 2020), in addition to the anxious symptoms with greater centrality of the first wave, item A3 (overconcern) and item A2 (lack of control of worry) also stood out, which were more connected to each other. At the beginning of this period, obligatory quarantine was reinstated, and the elderly became the first to be isolated in the face of heightened infection alarm, in fact, over one million people had been infected in the UK (66). In this confinement, older adults were again more exposed to media and social networks that provided more fatalistic news related to the global pandemic (e.g., new variants of COVID-19), and were more aware of the perceived risk of death from the loss of a family member or friend (67). In addition, in early December, the UK started vaccinating the elderly in the UK as they were considered a vulnerable population (3), which triggered further distress due to existing controversies and conspiracy news about vaccination that reinforced further uncertainty and anxiety about possible side effects and lack of confidence about the benefits of the vaccine (68–70). Therefore, it was evident that older adults who were assessed during the second wave were still experiencing a tendency to worry in an exaggerated way and with difficulties in controlling worry. The latter anxious symptom has also been previously reported as one of the most central among interacting systems of anxious and depressive symptomatology in young and older adults during the pandemic (65, 71–73).

For the second objective, it is shown that the symptom of depressive state (D1) was the most directly and indirectly interconnected (bridging symptom) in both networks. These results are similar to other studies that considered psychological distress symptomatology in Chinese university students during the COVID-19 pandemic (71, 74, 75). Such a symptom may reinforce the influential comorbidity between the symptoms of the three communities, specifically it has a stronger direct connection with the symptom of lack of control over worry (A2) and exclusion (L2). People who feel socially excluded often experience negative emotions to the point of triggering depressive symptoms, such as feelings of sadness, anger, and decreased self-esteem (76–78). In addition, all of these symptoms increase worry about daily activities, which leads to an increase in symptoms of generalized anxiety. Several network studies during the pandemic reveal an association between these anxious symptoms and an increased depressive state (65, 74), indeed, a recent study by Tao et al. (71) indicates that lack of control over worry and depressed mood are more influential in the manifestation of psychological distress symptomatology in later stages of the pandemic. Studies have reported that adults older than 50 years report greater physical health problems, especially those with a diagnosis of depression (79). People with these comorbidities may present somatic and physiological symptoms that reinforce a greater degree of fatigue, lack of energy, and insomnia (80).

Another of the common results of both networks is the recognition of feeling lonely (D5), which is intertwined between the domains of depression and loneliness. Although this indicator is of an emotional nature, it is more closely related to symptoms of social loneliness, especially lack of companionship (L1). Other network studies found that perceived loneliness was more strongly related to depressive state in adults residing in Switzerland (81), while Feiten et al. (82) found that social withdrawal is connected with the emotional state of sadness. Given that high levels of loneliness have been reported during the first two waves of confinement in the UK in conjunction with depressive symptoms, this may reflect that the loss of social connectedness linked to social loneliness leads to less social engagement. Indeed, this interpersonal distancing often persists after the confinement state as a state of chronic loneliness (83) in the face of increased perceived social isolation, which has been shown to contribute to higher rates of morbidity and mortality (84, 85). It is especially of greater interest in older adults who find themselves with little social companionship, as loneliness is related to some harmful health behaviors such as poor diet, physical inactivity, alcohol consumption, and smoking (86).

In the first network, it is identified that the unhappiness measure (D4) is intertwined in the three network communities. This finding using the Clique Percolation method has been used in previous studies where symptoms and feelings interact which may be intertwined (87, 88) and partially aligns with another previous finding that identified the cross overlap of the symptom of sadness (unhappiness) between depression and anxiety in psychiatric patients (87). The network results obtained are more reliable compared to previous work with overlapping psychopathological symptomatology network that reported negative relationships between anxiety and depression symptoms, and even some symptoms did not correspond to any symptomatological community (26, 87).

In this first network it is also recognized that a greater number of symptoms are identified with the anxiety domain, which includes the symptom related to sleep problems (D3). Within the DSM-5 Mental Disorders Manual, such symptom is recognized as one of the diagnostic criteria for generalized anxiety disorder (GAD) (89). Therefore, it can be considered as a transdiagnostic symptom and of greater anxious impact produced by the first months of mandatory quarantine in older adults (90), which is more closely linked to difficulty relaxing (A4; higher expected influence and anxiety/depression overlap) in the first network. These findings are in line with previous studies pointing to the interaction of such symptoms in the network (26, 87), even in network outcomes where such symptoms are central (72, 91).

Additionally, in the second network we identified that the symptom of sleep difficulties (D3) presented a cross grouping within the structure of anxiety and depression, which indicated its more comorbid manifestation during the period of the second quarantine. Insomnia and difficulty initiating sleep have been reported to be related to symptoms of worry and depression in older adults (82). This finding has been evidenced in studies conducted in the last quarter of 2020, after the first wave, where the symptom of sleep problems was identified as the most interconnected measure in psychometric networks comprised of symptoms of anxiety, insomnia, and depression (62, 91).

Another difference in this network, was the unique membership of the unhappiness symptom (D4) in the depression community. This was likely a result of the re-implementation of lockdown measures, leading to increased feelings of dissatisfaction brought about by renewed social isolation and a perceived loss of freedom (92). This outcome was strengthened because the occurrence of unhappiness was linked with depressive emotions (D1). In this way, it could support previous research that highlights the connection between unhappiness and a depressive state in adults who had chronic conditions (93) and in pregnant Latina women (94).

On the other hand, we detected that the networks representing both waves showed a noticeable sequence of stronger and similar associative patterns: from the item of feeling lonely (D5), which reinforced the symptom of feeling depressed (D1), which interacted with other depressive indicators such as anhedonia (items D2 and D7) and difficulty to rest (D3), until connecting with the anxiety symptom of difficulty to relax (A4), which reinforced other anxious manifestations. It is likely that these findings denote a greater sense of emotional vulnerability that reinforced other psychophysiological symptoms more characteristic of anxiety in those older adults who felt a greater need for companionship during the COVID-19 quarantine period. This makes sense if we consider that this period originated disruptive events such as family estrangement and the adoption of new self-care health measures, events for which many older adults were not prepared to face, or even imply new burdens to pre-existing ailments or other difficulties (95, 96).

Finally, for the third objective, we identified the longitudinal network that included the variables during the two pandemic waves, we observed a clear influence directed toward the anxious symptom of nervousness (A1), mainly from the depressive symptom of difficulties to enjoy life (D6), which activated both the loneliness items about feeling excluded (L2) and apart from others (L3), and these in turn had a stronger direct effect on the nervousness symptom. These effects are similar to other reports that indicate that the prolongation and typical limitations of quarantine made the increase of symptoms linked to anhedonia more likely (97), while this state of low motivation propitiated a greater sense of helplessness, to the point of having the sensation of being alone or remaining distant from others (98).

### Limitations

On the other hand, it is necessary to mention some limitations. Although we examined variables relevant to mental health in a vulnerable population, future research should take into account other aspects such as socioeconomic, cultural, or relevant physical health history to contrast our results using the same available database. Moreover, although the information was collected during a critical period for the population, it is necessary to continue evaluating the study variables even during the context of new variants of COVID-19 or other viruses (e.g., monkeypox). This would be useful to verify whether the interactions found were situational, whether they maintain their level of severity or are part of a more generalized phenomenon over time. Despite these limitations, we consider that the findings of the present study contribute to a plausible explanation of the reactions obtained in older adults in a context of constant threat to their physical and psychological health. This allows establishing preventive measures in the medium term, especially when it comes to the importance of the necessary support (family, friends, and neighbors) to face adverse experiences, the learning of virtual communication in future similar events and the usefulness of promoting regulatory resources of emotions, including recreational activities at home.

## Conclusion

In summary, it was evident that for the June-July stage network, anxiety symptoms were of greater centrality, where item A4 (difficulty in relaxing) and item 1 (nervous or anxious) stood out. Moreover, the network analysis of the second wave (November–December) found that in addition to the anxious symptoms with the highest centrality of the first wave, item A3 (overworrying) and item A2 (lack of control of worry) also stood out, which were more connected to each other. Similarly, the symptom of depressive state (D1) was reported to be the most directly and indirectly interconnected symptom (bridging symptom) in both networks. Another common finding in both networks is the cross-identification of the symptom of feeling lonely (D5) in the depression and loneliness domain. Finally, when identifying the longitudinal network that included the variables during the two pandemic waves, we observed a clear influence directed toward the anxious symptom of nervousness (A1), mainly the depressive symptom of difficulties in enjoying life (D6), which activated both the loneliness items about feeling excluded (L2) and apart from others (L3), and these in turn had a stronger direct effect on the nervousness symptom.

## Data availability statement

Publicly available datasets were analyzed in this study. This data can be found at: https://www.elsa-project.ac.uk/.

## Ethics statement

Ethical review and approval was not required for the study on human participants in accordance with the local legislation and institutional requirements. The patients/participants provided their written informed consent to participate in this study.

## Author contributions

CR-V and AG contributed to the conceptualization, formal analysis, and drafting of the manuscript. MD and DH assisted in data curation and preparation of the original draft. JS have contributed to the research and writing of the manuscript. All authors have read and accepted the published version of the manuscript.
